# OPLE: Drug Discovery Platform Combining 2D Similarity with AI to Predict Off-Target Liabilities

**DOI:** 10.3390/ph19020228

**Published:** 2026-01-28

**Authors:** Sarah E. Biehn, Juerg Lehmann, Christoph Mueller, Fabien Tillier, Carleton R. Sage

**Affiliations:** 1 Eurofins Discovery Services North America, LLC, 6 Research Park Drive, Saint Charles, MO 63304, USA; sarah.biehn@discovery.eurofinsus.com (S.E.B.); fabien.tillier@discovery.eurofinseu.com (F.T.); 2 Beilstein Institut, Trakehner Strasse 7-9, 60487 Frankfurt am Main, Germany

**Keywords:** machine learning, molecular similarity, off-target safety, Tanimoto similarity, extended-connectivity fingerprints, safety pharmacology

## Abstract

**Background/Objectives**: An impediment to successful drug discovery is the potential for off-target liabilities to eliminate otherwise promising candidates. As the drug discovery process is time-consuming and expensive, the use of artificial intelligence (AI) methods such as machine learning (ML) has drastically increased. It is invaluable to generate models that can quickly differentiate between successful and unsuccessful small-molecule drug candidates. Previous efforts established that molecular similarity could be used with other metrics to inform predictions of potential activity against a protein target. Similar methods were pursued here to combine similarity and machine learning for a collection of models called OPLE. **Methods**: Models were trained with proprietary and publicly available data to predict the likelihood of a given compound to be active against targets present in existing experimental SafetyScreen panels 18 and 44. Two-dimensional (2D) Tanimoto similarity from extended-connectivity fingerprints (ECFPs) and trained ML models were combined to obtain predictions. **Results**: Using all training data, a relationship between similarity and activity was established by fitting a probability assignment curve. Calibrated ML label assignment likelihoods were joined with the predictions from ECFP Tanimoto similarity to known active compounds using the belief theory formula, which maintains that activity prediction increases when both pieces of evidence support it. When assessing the performance of OPLE models for SafetyScreen 18 and 44 targets with external data from ChEMBL, more than 80% of the models had recall values greater than 0.8. This indicated favorable predictive ability to identify active molecules while limiting false negative predictions. **Conclusions**: Predicting and experimentally verifying safety liabilities is insightful at every stage of small-molecule drug discovery. This early detection tool can help project teams save resources that could be better deployed on series with no predicted or measured off-target liabilities.

## 1. Introduction

Small-molecule drug discovery, despite remarkable technological and methodological advances, continues to be a highly resource-intensive endeavor. The process of bringing a new therapeutic compound from initial concept to market approval typically takes a decade or more and can cost hundreds of millions of dollars. Each stage—target identification, hit discovery, lead optimization, preclinical testing, and clinical trials—requires substantial investment in both time and expertise. The high attrition rate of drug candidates, especially due to the safety or efficacy failures in late-stage development, further adds to these challenges. Therefore, identifying the presence of an off-target effect early in the exploration of a chemical series would be substantially useful.

In recent years, artificial intelligence (AI) has been increasingly applied to drug discovery, offering new strategies to address long-standing inefficiencies. Machine learning (ML) models, a subset of AI, are particularly promising. By leveraging large, multidimensional datasets, such as chemical structures and biological assay results, ML can be used to identify patterns that may be indiscernible to traditional heuristic or rule-based approaches.

ML models cannot replace experimental validation. Despite this, ML models can provide valuable guidance for prioritizing compound series, predicting biological activity, and assessing potential liabilities, especially when reasonable confidence values for predictions can be proscribed. Such usage aids in the pursuit of trying to save effort, cost, and, most critically, time across multiple phases of discovery.

A crucial aspect of small-molecule drug discovery includes the assessment of whether candidate compounds will interact with proteins known to perpetuate off-target safety concerns. Adverse effects arise when unintended interactions occur between the potential drug and known protein safety targets, which can ultimately cause the discontinuation of otherwise promising drug candidates. One such example is fenfluramine. Although quite efficacious for weight loss, fenfluramine was later found to be an agonist at the serotonin 2B receptor. This off-target interaction is responsible for cardiac valvulopathy and has been associated with pulmonary arterial hypertension, both of which can ultimately lead to heart failure. As a result, the drug was withdrawn from the market in 1997 [1]. Examples such as this reiterate the importance of systematic evaluation of compounds for potential off-target liabilities early in development.

To mitigate similar risks, researchers rely on standardized in vitro safety pharmacology panels such as SafetyScreen 18, SafetyScreen 44, SafetyScreen 59, SafetyScreen 89, and BioPrint panels. These panels evaluate off-target potency for an increasing number of protein receptors and ion channels. In some cases, regulatory agencies or internal governance policies might even require such assessments before the compounds can progress to animal studies or clinical trials. Regulatory agencies such as the FDA and EMA generally expect safety assays to be conducted prior to clinical trials, although the only formally required assay is the hERG inhibition assay to assess cardiac risk [2,3].

Given the cost and time associated with experimental safety testing, interest in computationally predicting safety panel outcomes continues to grow. Previous works have sought to computationally predict safety panel performance [4,5,6,7,8,9,10,11,12,13,14]. While ML models are relatively quick to build and easy to use, such models might be limited to singular conventional methodologies to train models.

One effort to predict activity likelihoods by Muchmore et al. found that using a combination of fingerprint similarity along with 3D shape similarity led to accurate predictions of compound potency [15,16]. That work served as a basis for this present work, as it was hypothesized that potency likelihood informed by multiple methodologies would promote accurate predictions. Inspired by the Muchmore effort, the Oracle for Protein/ligand binding Liability Evaluation (OPLE) was built to expand the Eurofins DiscoveryAI suite of predictive capabilities [17]. We sought to develop a novel methodology by combining 2D similarity with extended-connectivity fingerprints (ECFPs) with the addition of ML models to predict potential off-target liability against safety panel targets [17,18,19,20].

In this work, we provide an overview of our effort to produce OPLE models. OPLE was trained with a combination of proprietary and publicly available data containing over 133,500 molecules, including 20,000+ active molecules, across 40-plus safety protein targets. As in Muchmore et al., a relationship between fingerprint-based similarity and fraction of molecules displaying activity was established globally with data for all protein targets. From there, OPLE models employed similarity to known active molecules in combination with calibrated ML model probability predictions to deliver a robust framework. Models in the OPLE application provide users with the percentage likelihood of being active against safety targets, applicability domain information, and the top nearest neighboring compound from the active compound library. Because of the way OPLE was designed and trained, it is best served as a tool to identify compounds with high similarity to known active compounds. Overall, OPLE is an easy-to-use tool to assess potential liability for users’ project-specific needs.

## 2. Results and Discussion

### 2.1. OPLE Active Library Contains over 20,000 Unique Compounds Across 40+ Targets

OPLE models were trained in over 133,500 molecules from a combination of proprietary and publicly available data. Datasets included the simplified molecular-input line-entry system (SMILES) representations of chemical compounds along with the corresponding experimental assay values measured as IC_50_. Proprietary data originated from EMERALD, a collection of experimental data collected during the BioPrint project in the early 2000s [19]. The BioPrint database features approximately 2500 compounds tested in a multitude of target-based assays. Roughly 60% of the compounds were marketed drugs, while the remaining percentages split across other compounds, such as compounds that were clinically tested but not marketed, withdrawn drugs, standard references, food additives, veterinary drugs, and so on. The BioPrint data provided a quality mix of not only successful drugs (marketed drugs) but also unsuccessful candidates (withdrawn and tested but not marketed drugs), making it a valuable dataset for model training. Additionally, the EMERALD data was enriched with publicly available data from the BindingDB, a publicly curated collection of protein–ligand data [18]. The EMERALD data took precedence and was retained during data curation efforts because of the quality and reliability of the dataset. The BindingDB data supplementing the OPLE training sets provided a rich chemical space context that expanded OPLE active libraries for nearly every protein target, increasing the applicability domain of OPLE models.

**Figure 1:**
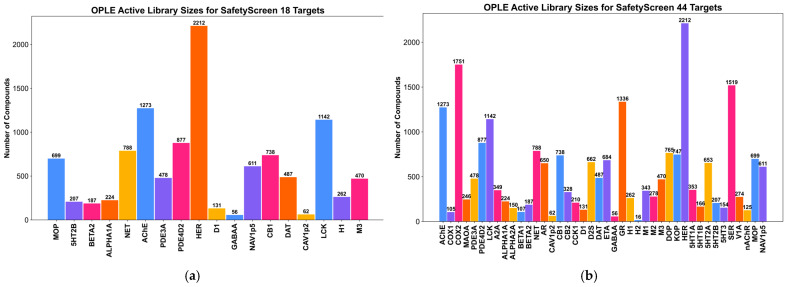
Number of active (IC_50_ ≤ 100 nM) compounds within each safety target active library in (**a**) SafetyScreen 18 OPLE models; (**b**) SafetyScreen 44 OPLE models. Each bar represents the active library size for one OPLE model for the labeled protein target. Active libraries ranged in size from 16 to 2212 molecules. The average size for active libraries ranged between 400 and 500 molecules.

To make Tanimoto similarity comparisons using ECFP, each safety target required a collection of active compounds that could be compared to input compounds. In a less-strict active definition than Muchmore et al., active compounds were defined as molecules with a half-maximal inhibitory concentration (IC_50_) value less than or equal to 100 nanomolar (nM). While Muchmore et al.’s active threshold of 10 nM was initially pursued, we increased the active threshold to 100 nM to accurately capture clinically relevant high receptor affinity [15]. We did not pursue a micromolar affinity threshold due to weaker interactions that may or may not be clinically relevant. Overall, the 100 nM potency cutoff balanced legitimate risk of off-target safety effects with a better dataset balance. Active libraries were built using compounds from the proprietary Eurofins Discovery EMERALD database and from curated publicly available data from the BindingDB. The breakdown of the number of active compounds per safety target is shown in Figure 1 for SafetyScreen 18 models (Figure 1a) and SafetyScreen 44 models (Figure 1b).

Across all targets, the SafetyScreen 18 active library consisted of 10,405 compounds, while the SafetyScreen 44 library contained 18,950 compounds. Combined, the active library consisted of 20,019 unique compounds of which 1417 (7%) active compounds originated from the EMERALD database. This served as a substantial dataset from which OPLE models could be trained.

### 2.2. Global Probability Assignment Curve Based on ECFP Tanimoto Similarity

OPLE models were built by establishing a relationship between molecular similarity and likelihood of activity and by training machine learning models. OPLE model development is diagrammed in Supplemental Figure S1 and described in detail below.

**Figure 2:**
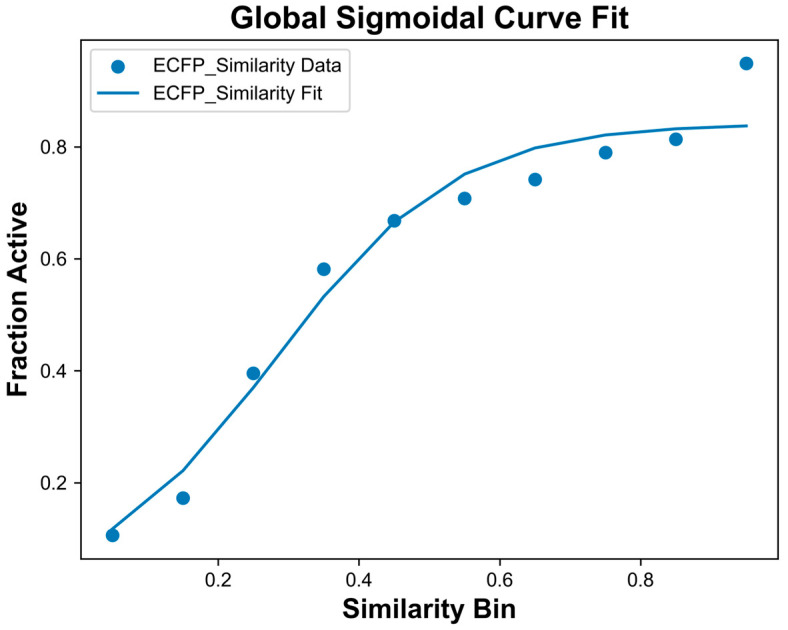
Probability assignment curve based on ECFP (radius = 6) Tanimoto similarity for all SafetyScreen 18 and 44 training data. Training data were broken into active, inactive, and random pairs for curve generation. Data points, shown as blue dots, were achieved by assessing the fraction of active compounds at each Tanimoto similarity bin between 0 and 1 with intervals of 0.1. The curve fit is shown as a solid blue line, and the equation of the fit was used for OPLE model predictions. The curve equation fit parameters were F_max_ of 0.842, SC_50_ of 0.281, and slope of 3.417.

To calculate the likelihood of activity based on Tanimoto similarity with ECFPs, the general protocol from Muchmore et al. was used, which found that ECFPs with a radius of six was one of the better predictive methods [15]. The idea centered around the probability assignment curve, which plotted ECFP Tanimoto similarity bins against the active fraction, or the ratio of active compounds to the total number of compounds in the similarity bin. Similarity bins were defined using 0.1 intervals between 0 and 1. This curve was generated through the creation of compound pairs using OPLE training data, as detailed in Muchmore et al. and summarized here [15]. Active pairs were defined as two molecules in which one compound was classified as active (IC_50_ ≤ 100 nM) and there was an IC_50_ difference less than one log unit, while inactive pairs were defined as two molecules in which one compound was classified as inactive (IC_50_ > 100 nM) and there was an IC_50_ difference between the two compounds greater than one log unit. Decoy pairs were also included in curve procurement, which consisted of pairings of one active molecule and one decoy obtained or generated using DUD-E [21]. The sigmoidal curve fit from the Muchmore work was based on all 23 targets for which they had data [15]. To test, pairs were initially used for probability assignment curve generation on a per-target basis. However, due to the wide discrepancy of dataset sizes across targets (detailed in Supplemental Table S1) and the better agreement with the original Muchmore methodology, the probability assignment curve used for all OPLE models was global, or based on all available pairs for all protein targets in SafetyScreens 18 and 44. The global probability assignment curve is shown in Figure 2, and all curve fit parameters were determined using global training data.

To create the probability assignment curve, active, inactive, and random pairs were grouped into similarity bins between 0 and 1 with 0.1 intervals. The fraction of compounds that were active in each bin was used to establish the relationship between similarity and belief of activity likelihood from ECFP Tanimoto similarity, or B_ECFP_. The relationship, or curve fit, allowed for the identification of three different fit parameters. The first term, F_max_, was the maximum fraction active value. The SC_50_ term, which can be viewed as conceptually similar to IC_50_, was the similarity threshold at which 50% of the maximum fraction active occurs. Finally, the equation has a slope parameter that assessed the steepness of the curve. Based on combining data for all available targets, the equation parameters were found to be F_max_ of 0.842, SC_50_ of 0.281, and slope of 3.417.

While these values differ from those reported in Muchmore et al. for ECFP_6 sigmoid curve parameters, we attributed these differences to variation in datasets and targets used. We suspected that the datasets in Muchmore et al. likely featured a high volume of series-specific data, typically including compounds that are structurally similar with minor modifications. As such, series data would have limited the chemical space coverage. The OPLE active compounds library, contrastingly, had a mean ECFP Tanimoto similarity value of 0.08 ± 0.02, indicating high diversity within the dataset. Furthermore, Muchmore et al.’s data was based on data for 23 different protein targets, whereas our OPLE dataset contained data for over 40 different protein targets [15].

### 2.3. ML Model Probability Calibration Efforts Were Explored to Improve OPLE Predictive Capability

To improve the predictive ability of OPLE, ML models were built for targets for which training data was available. As B_ECFP_ is the likelihood that a molecule is active based on ECFP Tanimoto similarity substituted into the probability assignment curve equation (as described in detail in the previous section), we hypothesized that we could use the predicted probability output from certain ML algorithms as an additional belief to inform predictions of the overall likelihood activity of an input molecule. Muchmore et al. used belief theory, or Hooper’s rule (Equation (2) in Section 3), which is the combination of independent beliefs to identify the likelihood that at least one belief supports the likelihood of occurrence. It was enacted in order to combine different methods of deducing likelihood of activity [15,16]. For our work, we prioritized using belief theory to combine B_ECFP_ and the likelihood, or belief, of activity predicted by ML models, B_ML_. Six different ML algorithms capable of returning a predicted probability were tested for each protein target, and the top-performing algorithm based on Matthews correlation coefficient (MCC) was pursued as the final target-specific model. MCC was the prioritized metric for model selection because it accounted for prediction quality considering dataset balance, which can be a pitfall of using accuracy alone. Most final trained models were XGBoost, random forest, or gradient boost, which was in alignment with algorithms that had historically been successful for experimental assay predictions [17]. The average accuracy and MCC of OPLE ML models are shown in Supplemental Figure S2. Initial performance targets were defined as at least 75% accuracy and MCC of 0.4, and OPLE ML models overall performed favorably [17,22]. Only three models performed under initial target thresholds, which we found could be attributed to small dataset size and/or dataset class imbalance, such as H2, which only had 16 active compounds.

While ML algorithms capable of providing probabilities of label predictions were used, the probability outputs from ML models were merely defined as relative confidence scores in class assignment. These outputs were not true probability. The outputs do not reflect the true likelihood of an event which requires calibration efforts to be achieved. Furthermore, the belief theory equation, or Hooper’s rule, assumed that the beliefs used were independent. B_ECFP_ was determined using fingerprints calculated from the SMILES. If the ML methods had been trained with fingerprints, B_ECFP_ and B_ML_ would not be independent, and the resulting belief theory result would be misused. OPLE ML models were trained using RDKit descriptors calculated from the SMILES, and it was important to grasp whether potential redundancy between beliefs occurred since both methods used the SMILES. To assess the dependence between B_ECFP_ and B_ML_ from models trained with descriptors, we examined the value of each belief and whether the ML-predicted outputs required calibration.

The redundancy between B_ECFP_ and ML probability outputs was investigating by assessing the Spearman ρ and mutual information, as shown in Supplemental Figure S3. These results reiterated the need for ML probability output calibration, as most models had higher Spearman ρ values indicating redundancy between beliefs. An analysis of OPLE ML predictions using different calibration techniques was performed by comparing belief combinations:

- B_ECFP_ determined from the probability assignment curve;
- The ML probability output (B_ML_);
- ML probability outputs that were sigmoid-calibrated (B_scML_);
- ML probability outputs that were isotonic regression-calibrated (B_icML_);
- The belief theory combination of B_ECFP_ and B_icML_;
- The combination of B_ECFP_ and B_icML_ with logistical stacking using interaction terms.

Sigmoid calibration involved fitting a sigmoid curve over raw ML probability outputs, while isotonic calibration was fitting a flexible, stepwise curve to map raw ML outputs to probabilities [23,24]. Logistical stacking with interaction used a trained meta-model that learned redundancy between B_ECFP_ and B_ML_ to weight each belief separately and together based on how much they agree with one another, with weights that rewarded or penalized the different terms based on their redundancy [25,26].

**Figure 3:**
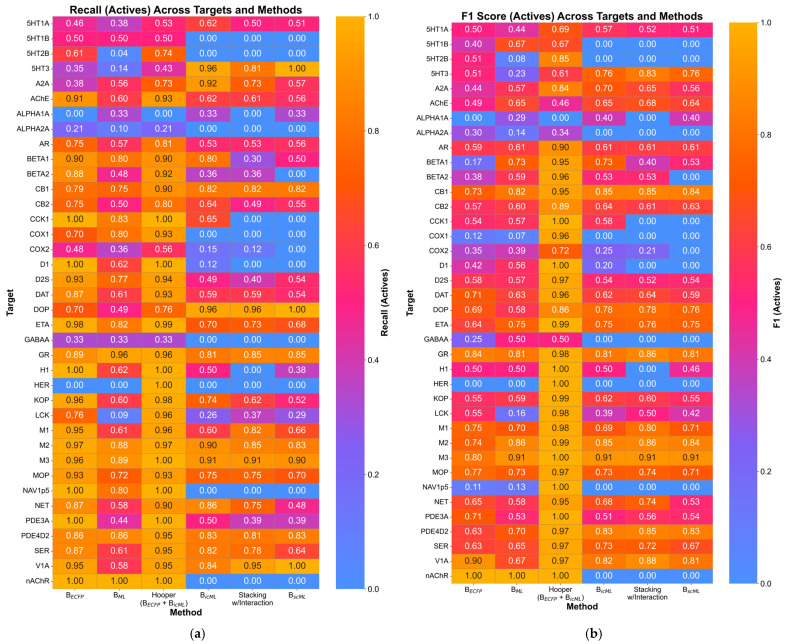
Heatmaps of (**a**) recall and (**b**) F1 score of active molecules from external test sets for each OPLE target when using different belief methods to predict activity. Values are listed on each cell. Cooler colors (blue, purple) indicate lower values while warmer colors (orange, gold) indicate higher values of recall or F1 score. As the metrics in this figure were calculated solely with active molecules, only targets with active molecules in the external dataset are included in the figure.

OPLE is best used as a tool to deduce whether input compounds are similar to compounds with known liabilities, so we prioritized our assessment with metrics that quantify performance based on correct active predictions. Precision, recall, and F1 score were calculated on predictions for available external test sets acquired from ChEMBL for each OPLE model. The most emphasis for favorable scores was placed on recall and F1 score. Recall, defined as the number of true positives divided by the sum of true positives and false negatives, was prioritized because it would be more favorable to minimize false negatives, or molecules that are active but are not truly predicted to be active. F1 score was examined as it balanced both precision and recall. The results are shown in Figure 3.

As seen particularly with recall, the combination of B_ECFP_ and B_icML_ tended to achieve the highest performance values. Most models (82%) had a recall value greater than 0.8, indicating favorable ability of OPLE models to minimize false negative predictions. This emphasized that isometric calibration of ML probabilities was the ideal path forward for incorporation into OPLE models. Furthermore, this finding validated the original Muchmore et al. findings that pursued the belief theory formula, or Hooper’s rule, to predict the likelihood of activity using multiple beliefs.

### 2.4. OPLE Predicts the Likelihood of Activity for Safety Panel Targets and Provides Nearest Neighboring Active Compound

**Figure 4:**
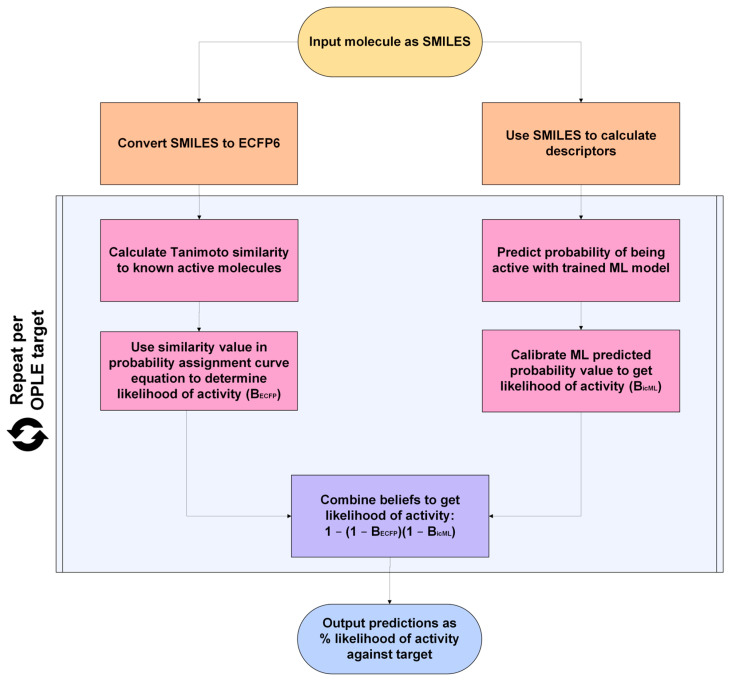
Flow chart diagramming OPLE predictive methodology.

The OPLE framework aims to predict the safety liabilities of user inputs and provide neighboring active compounds. OPLE empowers researchers to identify promising drug candidates with greater accuracy and to reduce the overall time and resources and invested in unsuccessful drug candidates. To provide the most value, OPLE is available through the Eurofins Discovery eCommerce platform to all users with an account who have ongoing Eurofins Discovery projects. The OPLE framework is depicted in Figure 4.

OPLE accepts input molecules as SMILESs, which are used in two ways. First, SMILESs are converted to ECFPs with a radius of six. ECFPs are used to calculate Tanimoto similarity of the input molecule to each known active in the OPLE active molecule library. For each target, the highest similarity value between a known active and the input is substituted into the probability assignment curve equation. The resulting value is B_ECFP_, which is the likelihood of activity based on ECFP Tanimoto similarity. Secondly, the input SMILESs are used to calculate descriptors, which are then used as inputs into the target-specific ML model. The ML model returns a predicted probability of active label assignment, which is then calibrated (B_icML_). B_ECFP_ and B_icML_ are fused using the belief theory equation to provide a joint prediction of the likelihood of activity. This result is returned as a percentage likelihood of activity to the user on a per-target basis. The top neighboring compound from the active library is supplied to the user as a nearest neighbor, providing additional insights.

To understand the relevancy of OPLE predictions, users should examine whether the input compound is within the applicability domain (reported with each prediction value) of OPLE models of interest. Users can establish their own applicability domain threshold by selecting a threshold value. The threshold value can be viewed as a Tanimoto similarity cutoff value. For example, if the applicability domain threshold is 0.5, the input compound will only be within the applicability domain of the model if the molecule has an ECFP Tanimoto similarity value of at least 0.5 to one of the compounds in the OPLE target’s active library collection. If no known actives for a target demonstrate a 0.5 ECFP Tanimoto similarity value to the input, the input compound is considered outside of the model’s applicability domain, and no prediction will be provided. A threshold of at least 0.7 is highly recommended.

Because of its molecular similarity approach, OPLE is most effective at identifying potential liabilities for compounds that have high similarity to known compounds with measured activity against targets that present known off-target activities. One obvious limitation of the OPLE tool is relative paucity of good target-based safety data. If a test compound is not similar to a compound for which a target-based safety liability is known, no reasoned prediction of the likelihood of a safety liability can be made. Ultimately, the choice to confirm potential experimental liability lies in the hands of end users. In addition, the lack of the identification of potential liabilities based on fingerprint or ML models is not an indicator that such a liability could not occur. Ligand/target interactions are manifold, and chemical space is vast.

## 3. Materials and Methods

OPLE models were trained with a combination of proprietary Eurofins Discovery EMERALD data and affinity data from BindingDB [18,19]. EMERALD data contained IC_50_ values for compounds with more than 30% inhibition in initial testing at 10,000 nM. Compounds below the 30% inhibition threshold were retained as inactive compounds. Public data were retrieved using the UniProt identifier of assay targets, and an IC_50_ upper limit of 10,000 nM was set for data retrieval. Final datasets were filtered to only include entries containing both SMILESs and IC_50_ affinity. Dataset sizes are detailed in Supplemental Table S1. Data were curated by implementing conventional quality inspections to remove duplicate SMILESs, to standardize SMILESs, and to validate SMILES stereochemistry and structure. Compounds were classified as active if the IC_50_ values were less than or equal to 100 nM, while compounds with IC_50_ values greater than 100 nM were considered inactive. For each target, DUD-E decoys were either retrieved from or generated based on active compounds using the DUD-E website [21]. Decoys were converted to SMILESs, which were used to generate ECFPs of the decoys to be used for probability assignment curve generation. External datasets for each target were collected from ChEMBL using UniProt identifiers listed on the Eurofins Discovery Services site [27,28]. Data were retained if the standard relations were equal (“=”) and the experimental assay values were listed as IC_50_ in nM. Any duplicate compounds already present in OPLE training sets were removed.

OPLE model generation centered on two components: the determination of probability assignment curve fit parameters from ECFP Tanimoto similarity and the training and calibration of ML models. Model generation was inspired by Muchmore et al. in which one of the top-performing similarity methods were ECFPs with a radius of 6 [15]. Probability assignment curve fit parameters were acquired by grouping training compounds into pairs based on their activity values then calculating the respective similarity values. Active pairs were defined as two compounds, with at least one of those compounds being classified as active, with an IC_50_ difference less than one log unit. Inactive pairs were two compounds, at least one of the compounds with an IC_50_ value greater than 100 nM, with an IC_50_ difference greater than one log unity. Random pairs consisted of one active compound and one decoy. While the DUD-E was built for docking benchmarking, the inclusion of comparing the similarity between actives and decoys during the probability assignment curve generation enabled a degree of structural separation in the dataset. The similarity values between the resulting pair sets were plotted against the fraction activity for all pairs, and the corresponding points were fitted to a sigmoidal curve equation as in Muchmore et al. [15]. The sigmoid curve equation is detailed in Equation (1):

(1) $$B_{ECFP}=\frac{F_{max}}{1+10^{({SC}_{50}-x_{i})\times slope}}$$

in which B_ECFP_ is the belief that two compounds will demonstrate the same level of activity, F_max_ is the maximum fraction active value, SC_50_ is the similarity threshold at which 50% of the maximum fraction active occurs, the slope is curve steepness, and x_i_ is the similarity value for compound pair i. ML models for SafetyScreen 18 and 44 targets were trained using RDKit descriptors, a 2D descriptor package that included terms such as logP, number of hydrogen bond donors and acceptors, number of heavyweight atoms, and so on [29]. Descriptors were pruned on a per-target basis by removing low-variance and highly correlated descriptors then subjected to a random forest pruning workflow. Six traditional ML algorithms in the Python (v3.12.4) package scikit-learn (v1.5.1) were explored with various hyperparameters, including weights applied based on the ratio between the number of active and inactive compounds in the training set [30]. ML algorithms that output probabilities for label assignments were prioritized [31,32,33,34]. The performance of each algorithm with tuned hyperparameters was evaluated based on repeated stratified sampling of five folds with three repeats per fold. Models were assessed based on accuracy, Matthews correlation coefficient (MCC), Cohen’s kappa, and area under the curve (AUC) metrics [35,36]. The trained method with the highest average MCC was selected, and the model with the highest MCC value was exported.

As probability predictions from ML models are relative assessments of confidence in class prediction, different probability calibration methods were tested. Five different variables were compared: the likelihood prediction from ECFP Tanimoto similarity (B_ECFP_), the raw probability output from ML models (B_ML_), sigmoid calibration ML probability outputs (B_scML_), isotonic calibration of ML probability outputs (B_icML_), logistical stacking with interaction of ML probability outputs (B_stackML_), and the combination of B_ECFP_ and isotonic-calibrated ML probabilities [23,24,25,26]. The previously trained ML models were used to train calibration models for each target using the scikit-learn module CalibratedClassifierCV by specifying the method parameter as “sigmoid” or “isotonic”. Equation (2) details the combination of beliefs, referred to as “Hooper’s rule” in the Muchmore et al. work:

(2) $$joint\;belief=1-(1-B_{ECFP})(1-B_{icML})$$

Methods were compared based on active predictions by assessing precision, recall, and F1 score [37,38,39,40]. Ultimately, combining beliefs according to Equation (2) and calibrating B_ML_ with isotonic calibration proved to yield the highest recall, and this method was employed in OPLE models.

## 4. Conclusions

Overall, OPLE provides an opportunity for users to predict safety liability likelihood based on molecular similarity and ML model predictions. As off-target liability predictions are valuable at each phase of the drug discovery process, OPLE adds value as an early warning system for off-target liability when similar compounds for the liability target are known.

## Data Availability

Publicly available data used in this study are openly available in the BindingDB at https://www.bindingdb.org. Proprietary EMERALD data were obtained and used with permission from Eurofins Discovery Cerep, to whom requests to access these datasets should be directed.

## Supplementary Materials

The following supporting information can be downloaded at: https://www.mdpi.com/article/10.3390/ph19020228/s1, Figure S1: OPLE model generation framework; Figure S2: ML model performance with metrics a, accuracy and b, MCC averaged over stratified test set splits; Figure S3: Spearman ρ (navy) and mutual information (MI, gray) values for each OPLE target based on the external ChEMBL test sets calculated prior to ML probability calibration. High Spearman ρ values suggest that BECFP and BML are redundant, while low ρ values suggest that there is value from combining beliefs. Higher MI indicated more dependence between beliefs. Results from this analysis emphasized the need to calibrate ML probabilities prior to using them in OPLE models; Table S1: OPLE training datasets.

## Abbreviations

The following abbreviations are used in this manuscript:

2D	two-dimensional
AI	artificial intelligence
B_ECFP_	likelihood of activity calculated based upon substituting the ECFP Tanimoto similarity into the probability assignment curve equation
B_icML_	likelihood of activity from isotonic-calibrated ML model predictions
B_ML_	ML probability outputs
B_scML_	likelihood of activity from sigmoid-calibrated ML model predictions
DUD-E	Database of Useful (Docking) Decoys—Enhanced
ECFP	extended-connectivity fingerprint
EMA	European Medicines Agency
EMERALD	Eurofins DiscoveryAI Exclusive Machine Learning Resource Leveraging Data as a Service
FDA	United States Food and Drug Administration
F_max_	maximum fraction active value
hERG	human ether-a-go-go-related gene
IC_50_	half maximal inhibitory concentration
MCC	Matthews correlation coefficient
ML	machine learning
nM	nanomolar
OPLE	Oracle for Protein/ligand binding Liability Evaluation
SMILES	simplified molecular-input line-entry system
