# Supplementary material for: OPLE: Drug Discovery Platform Combining 2D Similarity with AI to Predict Off-Target Liabilities
Source: Pharmaceuticals (Basel). 2026 Jan 28;19(2):228. doi: 10.3390/ph19020228 (PMC12943774; doi:10.3390/ph19020228)
Supplement: Supplementary file 1 [file pharmaceuticals-19-00228-s001.zip › pharmaceuticals-4035216-supplementary.pdf]

Supplemental Section

Supplemental Figure S1

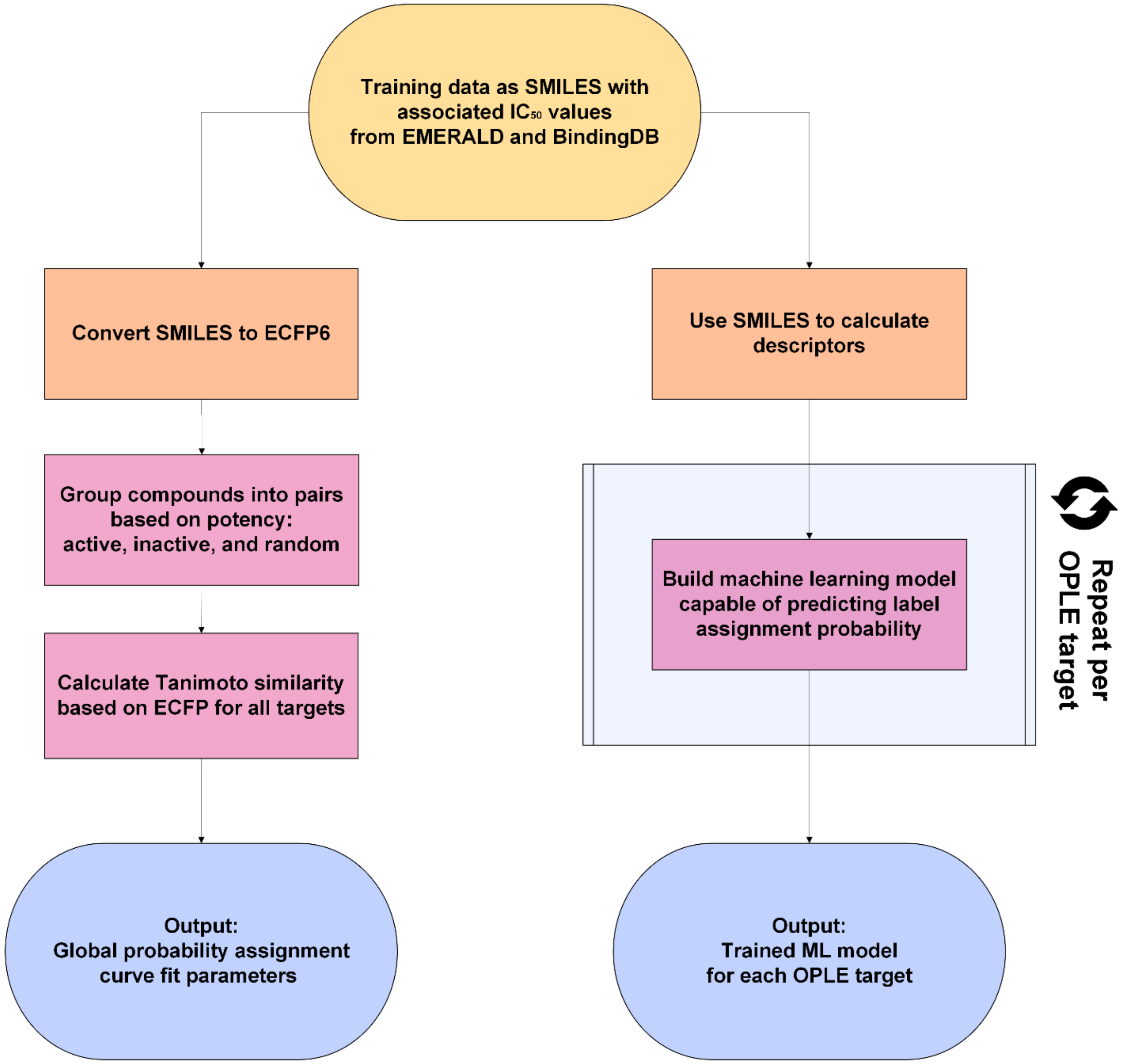

Supplemental Figure S1: OPLE model generation framework.

Supplemental Figure S2

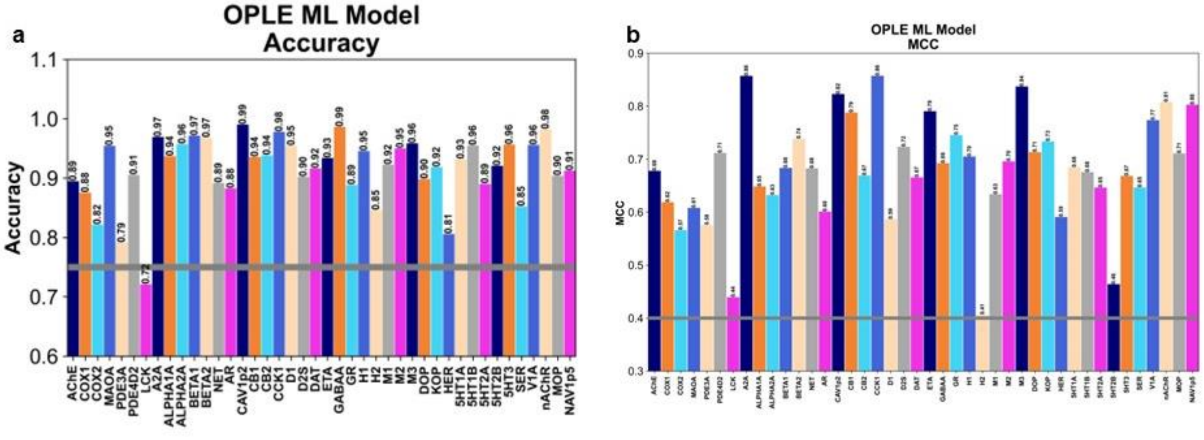

Supplemental Figure S2: ML model performance with metrics **a**, accuracy and **b**, MCC averaged over stratified test set splits.

Supplemental Figure S3

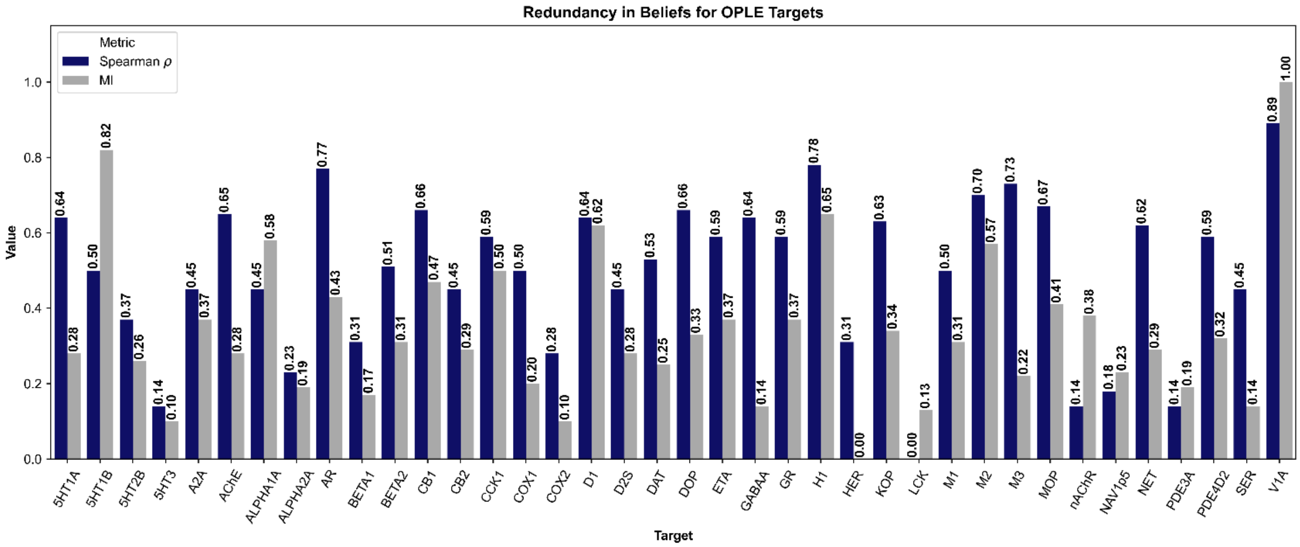

Supplemental Figure S3: Spearman ρ (navy) and mutual information (MI, grey) values for each OPLE target based on the external ChEMBL test sets calculated prior to ML probability calibration. High Spearman ρ values suggested that B_{ECFP} and B_{ML} are redundant, while low ρ values suggest that there is value from combining beliefs. Higher MI indicated more dependence between beliefs. Results from this analysis emphasized the need to calibrate ML probabilities prior to using them in OPLE models.

Supplemental Table S1: OPLE Training Datasets

OPLE Model Target	Training set size (source)	Percentage of active and inactive molecules
5-HT1A	2966 (18% BindingDB, 82% EMERALD)	12% active, 88% inactive
5-HT1B	2705 (10% BindingDB, 90% EMERALD)	6% active, 94% inactive
5-HT2A	3543 (31% BindingDB, 69% EMERALD)	18% active, 82% inactive
5-HT2B	2627 (7% BindingDB, 93% EMERALD)	8% active, 92% inactive
5-HT3	2705 (10% BindingDB, 90% EMERALD)	6% active, 94% inactive
A2A	2931 (17% BindingDB, 83% EMERALD)	12% active, 88% inactive
AChE	6228 (61% BindingDB, 39% EMERALD)	20% active, 80% inactive
ALPHA1A	2684 (10% BindingDB, 90% EMERALD)	8% active, 92% inactive
ALPHA2A	2716 (10% BindingDB, 90% EMERALD)	6% active, 94% inactive
AR	4429 (45%BindingDB, 55% EMERALD)	15% active, 85% inactive
BETA1	2877 (15% BindingDB, 85% EMERALD)	3% active, 97% inactive
BETA2	2942 (17% BindingDB, 83% EMERALD)	6% active, 94% inactive
CB1	3859 (37% BindingDB, 63% EMERALD)	19% active, 81% inactive
CB2	3454 (29% BindingDB, 71% EMERALD)	9% active, 91% inactive
CCK1	2776 (12% BindingDB, 88% EMERALD)	8% active, 92% inactive
COX1	3310 (26% BindingDB, 74% EMERALD)	3% active, 97% inactive
COX2	6947 (65% BindingDB, 35% EMERALD)	25% active, 75% inactive
D1	2673 (9% BindingDB, 91% EMERALD)	5% active, 95% inactive
D2S	3265 (25% BindingDB, 75% EMERALD)	20% active, 80% inactive
DAT	3906 (38% BindingDB, 62% EMERALD)	12% active, 88% inactive
DOP	4093 (40% BindingDB, 60% EMERALD)	19% active, 81% inactive
ETA	3596 (32% BindingDB, 68% EMERALD)	19% active, 81% inactive
GABAA	2502 (3% BindingDB, 97% EMERALD)	2% active, 98% inactive
GR	4465 (45% BindingDB, 55% EMERALD)	30% active, 70% inactive
H1	2726 (11% BindingDB, 89% EMERALD)	10% active, 90% inactive
H2	2495 (2% BindingDB, 98% EMERALD)	1% active, 99% inactive
KOP	4004 (40% BindingDB, 60% EMERALD)	19% active, 81% inactive
LCK	2077 (100% BindingDB)	55% active, 45% inactive
M1	3322 (27% BindingDB, 73% EMERALD)	10% active, 90% inactive
M2	3143 (22% BindingDB, 78% EMERALD)	9% active, 91% inactive
M3	3119 (22% BindingDB, 78% EMERALD)	15% active, 85% inactive
MAO-A	3842 (37% BindingDB, 63% EMERALD)	6% active, 94% inactive
MOP	3674 (34% BindingDB, 66% EMERALD)	19% active, 81% inactive
nAChR	2711 (10% BindingDB, 90% EMERALD)	5% active, 95% inactive
NET	4204 (42% BindingDB, 58% EMERALD)	19% active, 81% inactive
PDE3A	1079 (100% BindingDB)	44% active, 56% inactive
PDE4D2	4780 (49% BindingDB, 51% EMERALD)	18% active, 82% inactive
SERT	5175 (53% BindingDB, 47% EMERALD)	29% active, 71% inactive
V1A	2959 (18% BindingDB, 82% EMERALD)	9% active, 91% inactive
